# Using an accumulation of deficits approach to measure frailty in a population of home care users with intellectual and developmental disabilities: an analytical descriptive study

**DOI:** 10.1186/s12877-015-0170-5

**Published:** 2015-12-18

**Authors:** Katherine McKenzie, Hélène Ouellette-Kuntz, Lynn Martin

**Affiliations:** Department of Public Health Sciences, Queen’s University & Ongwanada, 191 Portsmouth Avenue, Kingston, Ontario Canada K7M 8A6; Department of Health Sciences, Lakehead University, 955 Oliver Road, Thunder Bay, Ontario P7B 5E1 Canada; Department of Public Health Sciences, Queen’s University, Kingston, Ontario Canada K7L 3N6

**Keywords:** Frailty, Intellectual disability, Developmental disability, Accumulation of deficits, Home care, RAI-HC, Aging

## Abstract

**Background:**

The aging population of adults with intellectual and developmental disabilities (IDD) is growing. In the general aging population, frailty is commonly used to predict adverse health outcomes, including hospital use, death, and admission to long-term care. However, existing frailty measures are less appropriate for aging persons with IDD, given their pre-existing conditions and limitations. An accumulation of deficits approach, which is now widely used to describe frailty in the general population, may be more suitable for persons with IDD. Frailty measures specific to persons with IDD have not been widely studied.

**Methods:**

Using pre-determined criteria, a frailty index (FI) specific to persons with IDD was developed based on items in the Resident Assessment Instrument - Home Care (RAI-HC), and using the assessments of 7,863 individuals with IDD in Ontario (aged 18–99 years) admitted to home care between April 1^st^, 2006 and March 31^st^, 2014. FI scores were derived by dividing deficits present by deficits measured, and categorized into meaningful strata using stratum-specific likelihood ratios. A multinomial logistic regression model identified associations between frailty and individual characteristics.

**Results:**

The resulting FI is comprised of 42 deficits across five domains (physiological, psychological, cognitive, social and service use). The mean FI score was 0.22 (SD = 0.13), equivalent to 9 deficits. Over half of the cohort was non-frail (FI score < 0.21), while the remaining were either pre-frail (21 %, FI score between 0.21 and 0.30) or frail (27 %, FI score > 0.30). Controlling for individual characteristics, women were more likely to be frail compared to men (OR = 1.39, 95 % CI: 1.23–1.56). Individuals who were frail were significantly more likely to have a caregiver who was unable to continuing caring (OR = 1.86, 95 % CI: 1.55–2.22) or feeling distressed (OR = 1.54, 95 % CI: 1.30–1.83). Living with a family members was significantly protective of frailty (OR = 0.35, 95 % CI: 0.29–0.41), compared to living alone.

**Conclusions:**

Using the FI to identify frailty in adults with IDD is feasible and can be incorporated into existing home care assessments. This could offer case managers assistance in identifying at-risk individuals. Future analyses should evaluate this measure’s ability to predict future adverse outcomes.

**Electronic supplementary material:**

The online version of this article (doi:10.1186/s12877-015-0170-5) contains supplementary material, which is available to authorized users.

## Background

Population aging and its associated challenges have been repeatedly reported by policy makers and health researchers across the globe [1]. Much of the aging research has focused on the general population; the subset with intellectual and developmental disabilities (IDD) has received relatively little attention [2].

Almost 1 % of the population has an IDD [3, 4], which the World Health Organization defines as “a group of developmental conditions characterized by significant impairment of cognitive functions, which are associated with limitations of learning, adaptive behaviour and skills” (page 177) [5]. These conditions originate before the age of 18 years [6], with the expectation of lifelong disability.The risk of health problems is greater among persons with IDD [7], and these contribute to the widely held notion that this group experiences premature aging, where 50 years may be considered “old” [8–12].

While many illnesses and functional impairments become increasingly prevalent with age, age by itself is an insensitive and non-specific predictor of health vulnerabilities [13, 14]. Frailty is frequently identified as an effective measure of health and vulnerability [13, 15]. While a consistently applied definition does not exist in the literature, frailty is generally viewed as age-related decline associated with higher risk of adverse health outcomes [16]. However, few studies have measured frailty in adults with IDD. A review of the literature revealed the existence of two research groups explicitly measuring frailty in this population, each using a different approach.

Brehmer and Weber (2010) created a frailty measure, the Vienna Frailty Questionnaire for Persons with Intellectual Disabilities – Revised (VFQ-ID-R) [17, 18], which captures changes in 34 symptoms across four domains (physical, psychological, cognitive, and social). The presence of frailty is indicated if: (1) symptoms are present in at least three domains, and (2) a minimum of six symptoms are identified overall. Persons are described as “pre-frail” if only one of these criteria is met. In their sample of 147 Austrian adults with IDD aged 20–72 years, 17.7 % were frail, 17.7 % were pre-frail, and the remainder was non-frail [17]. The small sample size, however, hampers the generalizability of results. In addition, the difficulty in applying the components in the VFQ-ID-R to other studies or databases further limits its use.

In the Netherlands, Fried et al.’s (2001) five frailty phenotype symptoms (weight loss, weakness, poor endurance and exhaustion, low physical activity and slowness [19]) were measured in 848 individuals with IDD over 50 in the Healthy Aging and Intellectual Disabilities (HA-ID) study cohort [20]. Here, persons were frail if at least three of the five symptoms were present, and pre-frail if one or two were present. Evenhuis et al. (2012) [20] reported that 13 % of their cohort was frail and 60 % was pre-frail. Approximately 11 % of their sample under the age of 65 years was considered frail, which is similar to the published prevalence estimates of frailty in the general Dutch population aged 65 years or older. The findings support the hypothesis that persons with IDD experience frailty earlier than the general population [20].

The frailty phenotype approach to measuring frailty is limited in that it focuses on physical limitations, which are known to be more common in individuals with IDD regardless of age [10]. Others have noted that the strong influence of low mobility on frailty phenotype scores may lead to misclassification in the general population [21]; this may also lead to misclassification among those with IDD [10], who have higher rates of mobility limitations across the lifespan.

In a follow-up study, the HA-ID research group used an accumulation of deficits approach [22] to measure frailty in 982 adults 50 years or more using formal care in the Netherlands [10]. The accumulation of deficits approach emphasizes the proportion of health deficits a person has, rather than the presence of specific symptoms. The mean frailty score was 0.27 (SD = 0.13), with an upper limit of 0.69 (on a continuum ranging from 0 to 1, with a higher score representing frailty), and approximately 66 % of the sample was frail (score above 0.20). They also found that individuals with IDD had similar rates of frailty at age 50 as did the European population at age 70, again supporting previous work showing that persons with IDD are frail at earlier ages than in the general population.

While these are the only studies reporting on frailty measures developed specifically for populations of persons with IDD, generic frailty measurements have also been applied to this population. For example, in Ontario, Canada, the Frailty Marker, derived from the Johns Hopkins University Adjusted Clinical Group (ACG) System, is used to identify frailty in population-based studies relying on administrative data [23]. This marker categorizes individuals as frail based on 81 diagnostic codes. As the actual diagnostic codes are unknown (i.e., not published), it is suspected that the Frailty Marker relies on the presence of specific medical conditions and fails to capture the multiple domains required for a well-balanced frailty measure. As such, it may not be the most appropriate measure of frailty, which limits its use for both individual-level and population-level health service planning – especially among persons with IDD.

The objective of this study is to describe the process of applying the accumulation of deficits approach to develop a frailty index (FI) based on administratively held clinical data for use among community-dwelling adults with IDD receiving home-based health care services (i.e., home care), and to identify individual characteristics associated with frailty. Using associations with 1-year admission to long-term care (LTC), appropriate cut-off scores for the newly developed FI are determined, and the prevalence of frailty in this population is described.

## Methods

### Study design

This study is part of a larger program of research focused on Health Care Access Research and Developmental Disabilities (H-CARDD) (see www.hcardd.ca). In this study, the cohort includes 7,863 individuals living in Ontario in 2009/10, identified in one or more administrative health datasets (such as physician claims or hospital visits) as per a previous study [4, 23] and who had at least one home care assessment between April 1^st^, 2006 and March 31^st^, 2014. Individuals were between the ages of 18 and 99 years in 2009/10 and at their first home care assessment. The study protocol was reviewed and approved by the institutional review board at Sunnybrook Health Sciences Centre, Toronto, Canada, and the Queen’s University Health Sciences Research Ethics Board.

### Data linkage

The RAI-HC database contains the RAI-HC assessments provided by the Home Care Reporting System (HCRS). These data are stored with interRAI Canada and shared with the Institute for Clinical Evaluative Sciences (ICES). Data included the earliest home care assessment occurring in the study period for each cohort member. The Registered Persons Database (RPDB) contains information on all Ontario persons eligible for health coverage. For this study, it provided age (in years). The date of admission to LTC was retrieved from the Continuing Care Reporting System for Long-Term Care (CCRS-LTC) database, which provides demographic and clinical information about individuals receiving care in LTC homes. All accessed data had been de-identified using methods to ensure confidentiality and privacy. Datasets were linked using unique encoded identifiers and analyzed at the Institute for Clinical Evaluative Sciences (ICES).

### Dataset creation

In Ontario, home care services are provided by the Ministry of Health and Long-Term Care’s fourteen regional Community Care Access Centres, who determine eligibility and coordinate providers. Services provided include visiting health professionals, help from personal support workers, homemaking services and community support services. Case managers use the Resident Assessment Instrument- Home Care (RAI-HC) [24] as a needs assessment tool to assess those receiving, or about to receive, home care. InterRAI is an international collaboration that serves to collect and interpret data on health and social outcomes. The interRAI assessments, including the RAI-HC, have been implemented as routine and standardized measures in various care settings by several jurisdictions internationally, including several Canadian provinces [25]. The RAI-HC assessment captures information related to demographic characteristics, home environment, functioning, health, medications, informal support, and formal health services. With respect to demographic characteristics, the current study used age, sex, living situation (i.e., alone, with a spouse and/or child(ren), with other family, or in a group setting with non-relatives), residential care history (i.e., lived in a residential care facility in the last 5 years), and rural status. Following the Statistics Canada definition, rural status was defined as living in a “location not included in a [Canadian census metropolitan area or census agglomeration], living in an urban centre of fewer than 10,000, or living in a rural area” (p. 157) [26]. Rural status was determined from postal codes identified at time of assessment.

Following Brehmer and Weber (2010)’s [18] categorization of frailty domains, the health-related items of interest were categorized into cognitive, physiological, psychological, and social domains. An additional domain of “service use” captures other service-related items [10, 27]. The items for each domain were all selected from information available in the RAI-HC. Twenty-seven items related to cognitive patterns (e.g. memory loss, delirium), communication (e.g. ability to understand others), and practical skills (i.e., instrumental activities of daily living; e.g. help needed with ordinary housework, managing finances, shopping) were used to inform on the person’s cognitive functioning. One-hundred fourteen items were available to assess physiological health, such as: hearing, vision, bladder and bowel continence, health conditions (e.g. diarrhea, shortness of breath), nutritional status (e.g. morbid obesity, insufficient fluid intake), dental and oral status (e.g. chewing problems), skin conditions (e.g. pressure ulcers, wound care required), medical diagnoses (e.g. hypertension, Alzheimer’s disease, hip fracture, diabetes), and medications (e.g. use of anxiolytics, medication compliance). Psychological status was informed by seventeen items related to mood (e.g. feelings of sadness or depression, repetitive anxious complaints) and behaviours (e.g. wandering, verbal abuse). Items from the social domain included social isolation, withdrawal from activities of interest, and five other items.

In addition to these domains, items indicating home environment (e.g. difficulty accessing rooms in house, inhabitable heating/cooling) and service utilization (e.g. recent hospital admissions, unmet treatment goals) were used. Two global health status indicators were also accessed: self-reported health (asking if the individual feels he/she is in poor health) and the presence of diseases or conditions that make cognition, activities of daily living (ADL), mood, or behaviour patterns unstable.

Three variables related to informal support were included in this study: (1) caregiver is unable to continue caring (“caregiver inability”), (2) caregiver is unsatisfied with support from family and friends (“caregiver unsatisfied”), and (3) caregiver expresses feelings of distress, anger or depression (“caregiver distress”).

In addition to individual items, two measures embedded in the RAI-HC were included. Clinical Assessment Protocols (CAPs) identify common risks for individuals using home care, such as abuse, functional decline or LTC placement [28]. Algorithms using some RAI-HC items trigger CAPs, which then offer interpretations and potential interventions for case managers to include in home care planning [28, 29]. The Institutional Risk CAP is triggered for individuals with a high risk of institutionalization and suggests ways of remaining in the community [30]. The second embedded measure is the Cognitive Performance Scale (CPS), which provides the cognition level and characterizes individuals on a scale from 0 (intact cognition) to 6 (very severe impairment) [31]. Individuals with CPS scores ≥3 (i.e., moderate or worse cognitive impairment) were grouped into one category [32, 33].

### Analysis

#### Selection of health deficits

The RAI-HC assessment provided 180 deficit variables. Previous work has suggested that deficits can be signs, symptoms, disabilities, diseases or abnormal laboratory measurements [22]. The criteria for selecting variables, published first by Searle et al. (2008) [22] and then modified by Schoufour et al. (2013) [10] for persons with IDD, are described.

First, each deficit must be positively correlated with age. This was done by calculating Spearman’s correlation coefficients (r_s_) between each deficit (ordinal variables) and age (as a continuous variable). Deficits that were not significantly and positively correlated with age were excluded, using a cut-off of r_s_ = 0.05 (*p* < 0.0001). Second, deficits that were too saturated were excluded to prevent ceiling effects, using a prevalence cut-off of 80 %. For variables that were not dichotomous, deficits were considered present if any limitation existed. Third, the deficit must be associated with health status, which was determined using Chi-square tests (included if *p* < 0.05) for association. Fourth, a wide range of health aspects should be included in the FI. A review of the literature was conducted to determine if the deficits appeared to cover different aspects of health, including all five domains of health [18]. Searle et al.’s (2008) final criterion related to use of identical items over time is not relevant to this study, which assesses frailty at a single point in time [22].

Schoufour et al. (2013) developed further inclusion criteria for the FI that are appropriate for persons with IDD [10]. If a deficit variable has missing data for greater than 30 % of individuals, it should be excluded. Second, deficits were considered uncommon and excluded if prevalent in fewer than 5 % of individuals, to prevent floor effects. However, wherever possible, related variables were grouped to form multi-item deficits with a sufficient prevalence. Schoufour et al.’s (2013) [10] criteria also suggested reducing the number of variables if they appear repetitive. Variables were grouped into twenty-seven categories, and correlations between remaining variables within categories were determined. Variables that were very highly correlated (r > 0.9) were either grouped into a multi-item deficit, or only the item with the highest correlation with age was included.

The list of excluded variables was screened by experts (the authors) for deficits unexpectedly omitted. This current study added one further stipulation. In an attempt to identify a change in deficits, which is crucial to capturing frailty [14, 15], deficits were grouped whenever possible to create a “decline” variable to ensure that the FI included recent deficits, rather than long-standing functioning, health, or behaviour patterns.

#### Calculation of the continuous FI

Most variables were ordinal or dichotomous. Variables were recoded, if necessary, to scores of 0 (deficit not present), 0.5 (intermediate deficit), or 1 (full deficit present). One continuous variable (“falls frequency”) was recoded into an ordinal variable as 0, 0.25, 0.5, 0.75, and 1.0. The rescaling of deficits was congruent with previous publications [34, 35], although some expert judgment was required. Variables were not weighted, therefore all selected deficits contributed to the final FI score equally [36].

A FI score was calculated for each individual by dividing the sum of the deficit scores by the number of deficits measured, to create continuous values between 0 (no deficits present) and 1 (all deficits present).

#### Categorizing the FI

The FI can be informative as a continuous variable to describe and contrast populations’ distributions of vulnerability. However, to ease comparisons, the FI is often categorized into meaningful groups though cut-offs have not been consistently applied across studies [34]. Hoover et al. (2013) [34] reported methods to validate cut-off points for the FI, using stratum-specific likelihood ratios (SSLRs), to distinguish between frail and non-frail seniors and to identify “natural” ranges of frailty associated with different risks of adverse outcomes. SSLRs represent the likelihood that individuals in a specific frailty group (stratum) will experience an event (admission to LTC in 1-year follow-up) relative to their likelihood of not experiencing an event [34, 37]. Using a subset of the cohort (*n* = 7,115) with a home care assessment between April 1^st^, 2006 and March 31^st^, 2013, SSLRs were calculated. SSLRs are independent of the population prevalence [34], and are less susceptible to spectrum bias (i.e. change in measure characteristics due to a different mix of severity) than a single cut-off. This process helps to ensure that lower and higher scores are correctly assigned to their own corresponding group [38].

The ten stratum cut-off points identified by Hoover et al. (2013) [34] were used in this study. Strata were collapsed if there was an insufficient number of events or non-events, or if 95 % confidence intervals clearly overlapped [39]. Confidence intervals were calculated using equations presented by Peirce and Cornell (1992) [37]. This process ensured that strata were significantly different from each other.

#### Statistical analysis

The mean, standard deviation, and the maximum and minimum scores for the continuous FI are reported. A histogram shows the distribution of FI scores. Goodness-of-fit tests (e.g. the Cramer von- Mises test) assessed whether the distribution fit a Weibull or gamma distribution [40, 41]. The mean FI score per year was estimated by calculating the regression coefficient β [10], and the relationship between the upper limit of the FI and age was determined by plotting the 99^th^ percentile of each 10-year age group. The slopes of these scores can indicate the presence of an age-invariant sub-maximal limit to the FI, demonstrating that even with advancing age, no further deficits are accumulated.

Bivariate multinomial logistic regression models were completed to calculate odds ratios and 95 % confidence intervals to report the odds of frailty (pre-frail or frail compared to non-frail), by individual characteristics. These groups included age (per 10 year increase), sex, rural status, caregiver status variables (i.e. caregiver inability, caregiver unsatisfied, caregiver distress), living situation, residential care history, cognition level and the Institutional Risk CAP.

An adjusted multinomial logistic regression model was developed to determine adjusted odds ratios of frailty (pre-frail or frail compared to non-frail) and individual characteristics (listed above). Using backwards elimination to select significant covariates, at a significance level of α = 0.05 using the Wald test, the model retained significant covariates.

An analysis of variance (ANOVA) was completed to compare frailty groups by age (continuous). The correlation between frailty and self-reported health was determined. Bivariate logistic regression models were also developed for each individual deficit in the FI and 1-year admission to LTC; odds ratios and 95 % confidence intervals were determined.

All tests were two-sided tests, with an alpha value of 0.05, unless otherwise stated, to indicate statistical significance. All analyses were done using SAS Enterprise Guide version 6.1.

## Results

### Participant characteristics

In the study cohort, 52 % are female, and 17 % are living in a rural area (Table 1). The mean age of the sample at the first home care assessment is 56.2 years (median = 57 years). Twenty-two percent (22 %) of individuals reportedly lived alone, 17 % lived with a spouse and/or child(ren), and 35 % lived with other family members. A further quarter (25 %) of individuals lived in a group setting with non-relatives (e.g. a group home).

**Table 1:** Cohort characteristics

	*n*	%
Sex		
Female	4105	52.2 %
Male	3758	47.8 %
Rural status (*n* = 7,834)		
Rural	1337	17.1 %
Urban	6497	82.9 %
Caregiver inability		
Yes	1006	12.8 %
No	6857	87.2 %
Caregiver unsatisfied		
Yes	254	3.2 %
No	7609	96.8 %
Caregiver distress		
Yes	1270	16.2 %
No	6593	83.8 %
Living situation (*n* = 7,862)		
Lives alone	1733	22.0 %
Lives with spouse and/or child(ren)	1337	17.0 %
Lives with other family	2769	35.2 %
Lives in group setting	2023	25.7 %
Residential care history (*n* = 7,862)		
Yes	647	8.2 %
No	7215	91.8 %
Cognition level (CPS) (*n* = 7,861)		
Intact (0)	1086	13.8 %
Borderline intact (1)	1232	15.7 %
Mild impairment (2)	1416	18.0 %
Moderate + impairment (3+)	4127	52.5 %
CAP (institutional risk)		
Triggered	965	12.3 %
Not triggered	6898	87.7 %

### Development of the FI

#### Health deficits

The resulting FI includes 42 deficits across five domains (Additional file 1): physiological (*n* = 29, where 7 are related to ADLs and 10 to disease diagnoses), cognitive (*n* = 4), psychological (*n* = 3), social (*n* = 3), and service use (*n* = 2).

Only variables that met all of the criteria described were included as deficits in the FI (Additional file 2 provides the results of all items). One-hundred twenty-five deficits, such as smoking daily, bladder incontinence and multiple sclerosis, were excluded due to poor or negative correlation with age. Only one item was excluded due to a high prevalence (poor cognitive skills for decision-making), however sixty-seven variables did not meet the prevalence criterion (e.g. malnutrition, renal failure, hallucinations, vision decline). One variable, an instrumental activity of daily living (phone use), was excluded because over 30 % of the cohort had missing information or the activity did not occur.

Several items were pooled to create combined variables, including a general circulatory disease deficit that contained congestive heart failure, irregularly irregular pulse and peripheral vascular disease items. In addition, seven activities of daily living (ADL) variables were each combined with an ADL decline variable to create new items.

Some variables were highly correlated (e.g. pain intensity and pain frequency) (Additional file 3) and only one (i.e. pain frequency) was retained in the FI. No variables were deemed to be excluded inappropriately, due to the grouping of similar variables.

No participants had fewer than 39 variables measured: 85 % had complete data for all 42 deficit variables included, while the remaining 15 % were missing three deficits. The group with no missing deficits was compared to the group with missing deficits, and there were significant differences in frailty profiles (χ^2^ = 997, *p* < 0.0001). The group missing deficits were significantly more likely to be in the frail group (63 %) compared to the group with no missing deficits (20 %). The only deficits contributing to missing data were from the social domain.

The FI was more strongly associated with admission to LTC after 1-year (OR = 4.45, 95 % CI: 3.82–5.19) compared to the 42 individual deficits (Additional file 4). Nine deficits were not significantly associated with admission to LTC. The odds ratios of four deficits, all ADL decline items, were not significantly different than the FI’s odd ratio.

#### Characteristics of continuous FI

**Fig. 1:**
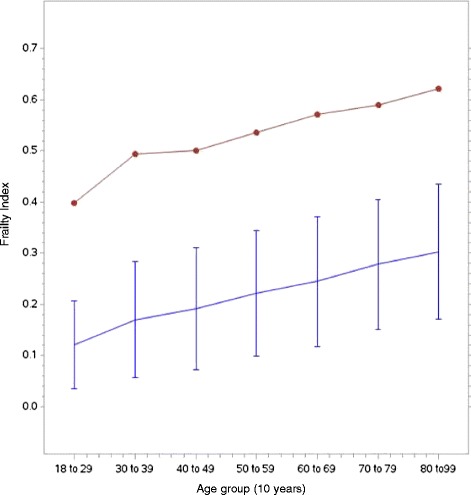
FI versus age plot: Average FI (bottom) and 99^th^ percentile of the FI (top), against 10-year age groups

The mean score of the FI was 0.22, with a standard deviation of 0.13, and a range of 0 to 0.77. Forty-eight (0.6 %) individuals were in a “zero state”, with no deficits present at all. The 99th percentile of the FI was 0.56, which is below the theoretical maximum of 1.0. The slope of age and the 99th percentile of the FI was significant (r = 0.49, (<0.0001), even in the subset of the cohort over age 50 years (r = 0.55, *p* < 0.0001) (Fig. 1).

**Fig. 2:**
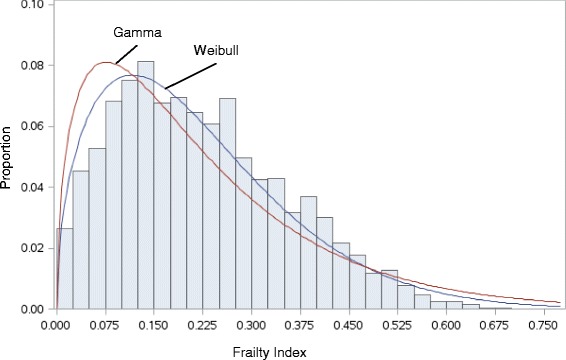
Distribution of the FI with a Weibull distribution curve and gamma distribution curve

For each increase of 1 year, the average change in mean of FI was +0.003 (+0.016 on a log scale), indicated by the average slope of deficit accumulation. The index had a right-skewed distribution (skewness value = 0.612), although the FI distribution was significantly different than both the gamma distribution (*p* < 0.001) and the Weibull distribution (*p* = 0.010) (Fig. 2).

#### Stratum-specific likelihood ratios

Initially, analyses used ten strata to categorize the FI, however not all strata had significantly different SSLRs when considering 1-year admission to LTC as the outcome (Table 2). Strata were combined to increase the power to detect differences between groups. The >0.10 to ≤0.21 stratum was significantly different than the previous stratum, identifying two distinct groups with likelihood ratios less than 1.0, which indicates a reduced risk of an adverse outcome. These strata were combined into a single, non-frail group (≤0.21).

**Table 2:** Stratum-specific likelihood ratios of 1-year admission to LTC with 95 % confidence intervals (*n* = 7,115)

			95 % CI			95 % CI	
FI strata	*N*	SSLR_10_	Lower	Upper	SSLR_3_	Lower	Upper
≤0.03	196	0.24	0.12	0.47			
>0.03 to ≤ 0.10	1199	0.29	0.23	0.37	0.50	0.45	0.55
>0.10 to ≤ 0.21	2301	0.63	0.56	0.71			
>0.21 to ≤ 0.23	367	0.98	0.74	1.29			
>0.23 to ≤ 0.25	445	1.02	0.79	1.30	1.05	0.93	1.19
>0.25 to ≤ 0.27	260	0.95	0.68	1.33			
>0.27 to ≤ 0.3	447	1.21	0.96	1.52			
>0.30 to ≤ 0.35	601	1.68	1.41	2.01			
>0.35 to < 0.45	844	2.14	1.87	2.45	2.21	2.05	2.38
≥0.45	455	3.21	2.69	3.84			

SSLR_10_ indicates SSLRs with 10 strata. SSLR_3_ indicates SSLRs with 3 strata

Further categorization suggests three frailty categories with significantly different risks of experiencing an event: non-frail (≤0.21), pre-frail (>0.21 to ≤0.30), and frail (>0.30). The frail group had a significantly higher likelihood of 1-year admission (SSLR = 2.21, 95 % CI: 2.05–2.38), while the pre-frail group had neither an increased nor decreased likelihood.

#### Prevalence of frailty

Over half (51.8 %) of the cohort was non-frail at the time of their first home care assessment. The remaining individuals were pre-frail (21.3 %) or frail (26.9 %) (Table 3).

**Table 3:** Cohort characteristics by FI group

	Non-frail		Pre-frail		Frail	
Overall (*n* = 7,863)	4073	51.8 %	1676	21.3 %	2114	26.9 %
Sex						
Female	1939	47.6 %	924	55.1 %	1242	58.8 %
Male	2134	52.4 %	752	44.9 %	872	41.2 %
Rural status (*n* = 7,834)						
Rural	681	16.7 %	270	16.2 %	386	18.4 %
Urban	3386	83.3 %	1399	83.8 %	1712	81.6 %
Institutional risk CAP						
Triggered	161	4.0 %	237	14.1 %	567	26.8 %
Not triggered	3912	96.0 %	1439	85.9 %	1547	73.2 %
Caregiver inability						
Yes	450	11.0 %	189	11.3 %	367	17.4 %
No	3623	89.0 %	1487	89.7 %	1747	82.6 %
Caregiver unsatisfied						
Yes	124	3.0 %	57	3.4 %	73	3.5 %
No	3949	97.0 %	1619	96.6 %	2041	96.5 %
Caregiver distress						
Yes	578	14.2 %	291	17.4 %	401	19.0 %
No	3495	85.8 %	1385	82.6 %	1713	81.0 %
Living situation (*n* = 7,862)						
Lives alone	680	16.7 %	486	29.0 %	567	26.8 %
Lives with spouse and/or child(ren)	432	10.6 %	386	23.0 %	519	24.6 %
Lives with other family	1894	46.5 %	427	25.5 %	448	21.2 %
Lives in group setting	1067	26.2 %	376	22.5 %	580	27.4 %
Residential care facility (*n* = 7,862)						
Yes	342	8.4 %	132	7.9 %	173	8.2 %
No	3731	91.6 %	1543	92.1 %	1941	91.8 %
Cognition level (CPS) (*n* = 7,861)						
Intact (0)	564	13.8 %	276	16.5 %	246	11.6 %
Borderline intact (1)	692	17.0 %	268	16.0 %	272	12.9 %
Mild impairment (2)	631	15.5 %	360	21.5 %	425	20.1 %
Moderate + impairment (3+)	2186	53.7 %	771	46.0 %	1170	55.4 %

The mean age was higher in the frail group (64.5 years) compared to the non-frail group (50.0 years) (*p* < 0.001). Frailty was positively correlated with poor self-reported health (r_s_ = 0.21, *p* < 0.001). Women were significantly more likely to be pre-frail (OR = 1.30, 95 % CI: 1.25–1.35) and frail (OR = 1.47, 95 % CI: 1.42–1.53) compared to men (Table 4). Living in a rural area, rather than an urban area, did not significantly change the odds of being pre-frail or frail over non-frail (*p* > 0.05). A prior admission to a residential care facility was significantly associated with reduced odds of being pre-frail or frail (OR = 0.60, 95 % CI: 0.47–0.76). Individuals who triggered the Institutional Risk CAP were much more likely to be frail compared to those who did not (OR = 9.19, 95 % CI: 7.47–11.31). This relationship between triggering a CAP and being pre-frail was also strong (OR = 4.32, 95 % CI: 3.45–5.42) (Table 4).

**Table 4:** Unadjusted and adjusted odds ratios of frailty and individual characteristics

Independent variable	Pre-frail vs. non-frail		Frail vs. non-frail	
	Unadjusted ORs (95 % CI)	Adjusted ORs (95 % CI)	Unadjusted ORs (95 % CI)	Adjusted ORs (95 % CI)
**Age (per 10 year increase)**	**1.42 (1.38–1.47)**	**1.30 (1.25–1.35)**	**1.63 (1.57–1.68)**	**1.47 (1.42–1.53)**
**Sex (female vs. male)**	**1.35 (1.21–1.52)**	**1.22 (1.08–1.38)**	**1.57 (1.40–1.74)**	**1.39 (1.23–1.56)**
**Rural status (rural vs. urban)**	0.96 (0.82–1.12)	-	1.12 (0.98–1.29)	-
**Caregiver inability (yes vs. no)**	1.02 (0.86–1.23)	1.13 (0.93–1.37)	**1.69 (1.46–1.96)**	**1.86 (1.55–2.22)**
**Caregiver unsatisfied (yes vs. no)**	1.12 (0.82–1.54)	-	1.14 (0.85–1.53)	-
**Caregiver distress (yes vs. no)**	**1.27 (1.09–1.48)**	**1.53 (1.29–1.83)**	**1.42 (1.23–1.63)**	**1.54 (1.30–1.83)**
**Living situation (ref. group = lives alone)**				
Lives with spouse and/or child(ren)	**1.25 (1.04–1.50)**	0.99 (0.82–1.19)	**1.44 (1.22–1.70)**	0.90 (0.75–1.09)
Lives with other family	**0.32 (0.27–0.37)**	**0.43 (0.36–0.51)**	**0.28 (0.24–0.33)**	**0.35 (0.29–0.41)**
Lives in group home	**0.49 (0.42–0.58)**	**0.53 (0.44–0.63)**	**0.65 (0.56–0.76)**	**0.59 (0.49–0.70)**
**Residential care history (yes vs. no)**	0.93 (0.76–1.15)	0.81 (0.64–1.02)	0.97 (0.80–1.18)	**0.60 (0.47–0.76)**
**Cognition level (CPS) (ref. group = intact)**				
Borderline intact (1)	**0.79 (0.65–0.97)**	0.94 (0.76–1.17)	0.90 (0.73–1.11)	1.08 (0.87–1.36)
Mild impairment (2)	1.12 (0.96–1.42)	1.18 (0.96–1.45)	**1.54 (1.27–1.87)**	**1.41 (1.14–1.75)**
Moderate + impairment (3+)	**1.23 (1.04–1.45)**	0.98 (0.81–1.18)	**0.72 (0.61–0.85)**	**1.46 (1.20–1.77)**
**Institutional risk CAP (triggered vs. not)**	**4.00 (3.25–4.93)**	**4.32 (3.45–5.42)**	**8.91 (7.40–10.71)**	**9.19 (7.47–11.31)**

Bolded values indicate significant at α = 0.05. Unadjusted ORs calculated from bivariate multinomial logistic regression models. Adjusted ORs calculated from one multivariate multinomial logistic regression model

Living with a spouse and/or child(ren) was not associated with being frail (*p* > 0.05) compared to living alone. Conversely, living with other family members or living in a group setting was protective against frailty (Family: OR = 0.35, 95 % CI: 0.29–0.41, Group Setting: OR = 0.60, 95 % CI: 0.47–0.76) compared to living in alone (Table 4).

Caregivers who reported that they were unable to continue caring had higher odds of caring for an individual who was frail compared to caregivers who did not report that they were unable to care (OR = 1.86, 95 % CI: 1.55–2.22). Being pre-frail did not appear to be associated with these caregiver feelings (OR = 1.13, 95 % CI: 0.93–1.37). Caregiver distress, however, was associated with increased odds of the individual with IDD being frail (OR = 1.54, 95 % CI: 1.30–1.83), and pre-frail (Table 4). Individuals with caregivers who were unsatisfied by the support from other informal caregivers were not at increased risk of frailty compared to those who were satisfied.

## Discussion

### Characteristics of the FI

This study applied an accumulation of deficits approach using deficits specific to a population of home care users with IDD. Frailty indices used in the general population may fail to identify items that are pertinent to this population because of a failure to account for life-long disabilities [10].

A FI was created using 42 RAI-HC variables. An index with a minimum of 30–40 deficits is reliable enough to predict adverse outcomes [42]. In the general population, mean FI scores derived using similar methods but different data sources and populations vary significantly: from 0.068 in a study of community-dwelling individuals aged 15–102 years [13], to 0.32 in an acute-care population of adults over 70 years old [35]. In Schoufour et al.’s (2013) [10] study of older adults with IDD living in the community (≥50 years), the mean frailty score was 0.27. The mean FI of the current study of 18 to 99 year olds was lower than that for Schoufour et al.’s (2013) [10] older population: a mean of 0.22 was calculated.

The FI had a right-skewed distribution [10, 22, 40]. Mitnitski et al. (2001) [41] reported that in populations that are “well”, the FI should follow a gamma distribution. Gamma distributions, and similar distributions (e.g. log-normal, Weibull), are often used for survival analysis and in frailty models [41]. The distribution of the FI in the current study followed neither a gamma nor a Weibull distribution, but it was right-skewed.

Another characteristic typically discussed is the presence of a sub-maximal, age-invariant limit. Typically, this limit is roughly two-thirds the maximum frailty score of 1.0 [22]. Sub-maximal limits indicate that the measure has no ceiling effects [43], which occur when a measure has a distinct upper limit for possible scores, and many participants are near this limit [44]. From a clinical perspective, a sub-maximal limit relates to the notion that an individual can be as frail as they could be, without experiencing every deficit or illness possible. This study did not find a sub-maximal limit where the slope of age and the 99^th^ percentiles of the FI became negligible. However, the maximum score reached was 0.77, indicating that very few individuals accumulated more than 30 deficits and survived in the community. While few individuals have high scores, typically even fewer individuals are in the zero state (i.e. a score of 0) as measured by a FI [45], which was also observed in the current study (<1 %).

The last characteristic of frailty indices frequently reported is the mean rate of deficit accumulation per year increase in age. While this rate varies, it is generally around 0.03 deficits per year on a log scale [42]. This rate is almost twice as high as the rate observed in the current study (0.016). Initially, this appears to give evidence against the hypothesis of premature aging in IDD. However, premature aging is not necessarily equivalent to fast aging. It could be that individuals with IDD begin accumulating deficits sooner, but more gradually. Rockwood et al.’s (2011) [13] relatively younger population of individuals (mean age 44 years) observed a slope of the line relating to the log mean of the FI of 0.029, almost double the slope observed in the current study. Unlike Rockwood et al. (2011)’s [13] findings, where a mean score of 0.21 was reached at 70 years of age, a mean score of 0.21 was reached in the mid-forties.

### Validation of the FI

Other research using this approach have validated the resulting measures of frailty across countries [42], ages [13], and health care settings [35, 46, 47], each potentially including different deficits following the same definition of frailty. However, as different and new deficits were selected for the current study, there is a need to consider and acknowledge the validity of the measure.

Rockwood et al. (2000) [14] specified that a measure of frailty should have: content validity (e.g. multisystem impairment, instability, changes over time, an allowance for heterogeneity within a population), construct validity (e.g. a positive association with aging), and criterion validity (e.g. a positive association with risk of adverse outcomes). Using this definition, the FI derived in this study appears to meet all the aspects defined.

To ensure that the measure considered multi-system impairment, deficits were categorized into five general domains (physiological, psychological, cognitive, social and service use) following guidelines in the literature. The number of dimensions of frailty vary [48–50], however Brehmer and Weber’s (2010) [18] categorization complies with the notion that frailty should include the environmental and social context of a person [14]. The FI created in the current study has deficits in all domains.

The definition of frailty also includes an element of instability. The derivation process ensured that the FI included items that captured a recent status change or decline wherever possible. The way a health deficit develops can be as critical, or even more critical, than the presence of the deficit itself [14, 51]. This may be especially true in persons with IDD, who likely have lower baseline health status due to life-long disabilities and higher predispositions to various health conditions [45].

Construct validity refers to whether the measure corresponds to similar measures and constructs [52]. Frailty should have some, although not perfect, correlation with age, co-morbidity and self-rated health [53]. In addition to a significant, positive association with age, the FI was positively correlated with poor self-reported health. A measure of co-morbidity was not included in the available data, but would have strengthened the construct validity of the FI.

Criterion validity is necessary to test a new measure against a reference. The measure should be compared to either a gold-standard test or evaluated based on its predictive ability. As there is no gold standard for frailty, a measure’s ability to predict adverse outcomes is the best method of validation. Adverse outcomes independently associated with frailty include, but are not limited to, mortality [54, 55], hospitalization [19] or falls [56, 57]. In this study’s adjusted model, the FI is significantly associated with age and admission to LTC. More work is required to understand the relationship between the FI and adverse outcomes, while considering relevant confounders.

In addition to assessing the validity of the FI, attempts were made to validate cut-off points for the FI. This study identified three frailty categories that can be used to distinguish between risk groups among persons with IDD. Defining three distinct groups, rather than four or five as in some other studies, may better distinguish between groups in future studies of adults with IDD with smaller sample sizes. While important information can be lost when forcing a single cut-off to create a dichotomous variable [39], three groups retain the potential to identify different risk groups. This was evident in the bivariate relationships presented in this study. For example, the caregiver inability variable had a strong relationship with the frail group, but is not strongly associated with the pre-frail group.

Using the FI, the 0.21 cut-off is widely used to discriminate between fit and frail in both the population with IDD [58] and in the population of Canadian seniors [13, 27, 34, 59]. Three frailty categories were identified. Hogan et al. (2012) [54] used similar cut-offs (<0.2, 0.2-0.3, >0.3) and labels. The “pre-frail” categorization is congruent with the literature: pre-frailty is an intermediate status that may indicate an increased risk of adverse outcomes [17, 19, 60].

Given that this FI specific to persons with IDD incorporates some items not included in previous studies, the potential impact of any given variable on the FI should be considered. Several studies have conducted re-sampling methods similar to “bootstrapping” by randomly selecting 80 % of deficits for inclusion in the FI and repeating this process for 1000 iterations. Using this strategy, authors have consistently reported negligible changes to the slope of the FI and age [10, 22, 35, 61] concluding that there is little sensitivity as to which deficits are included in the construction of the FI [35]. In other words, the proportion of accumulated deficits is more important than specific deficits. Given the consistency of these previous findings, this procedure was not repeated.

### Descriptive findings

Females had an increased risk of being pre-frail and frail. On average, women have more deficits than men, although they often tolerate these deficits better. Women have better rates of survival compared to men with the same level of frailty [10, 62, 63].

Frailty was strongly associated with caregiver status. Frail individuals require more care and daily assistance than pre-frail or non-frail individuals, and this may have some harmful effects on a primary caregiver’s well-being. Being pre-frail (rather than frail or non-frail) was not significantly associated with poor caregiver status, suggesting it is not until an individual accumulates roughly 12–13 deficits (FI score ≈ 0.3) that caregivers feel that they are unable to continue to care. Providing care for the frail elderly can lead to physical and mental health problems, and stress or burnout can increase the risk of admission to a LTC home for the person being cared for [64, 65]. Service providers should consider the resilience of families requesting services [66] to prevent crises that could lead to a move to a residential care facility. Additional research may wish to investigate if certain frailty deficits are larger contributors to caregiver distress than others.

### Strengths and limitations

Strengths of this study include the use of a standardized assessment instrument, the RAI-HC, as a source of data regarding deficits to create the FI. Items from the RAI-HC have strong correlations with their gold standard equivalents [67, 68], high internal consistencies (between 0.6 and 0.8) [69, 70] and high inter-rater reliability (mean κ = 0.69) [25]. The strength of the RAI-HC in this study is the wide range of items available, which provided deficits for all five domains of interest (physiological, psychological, cognitive, social, and service use). Compared to other datasets used to apply the accumulation of deficits approach to frailty measurement, the RAI-HC provided items that incorporated recent changes in health status, which is especially important among those with IDD.

This study has some limitations. It would have been informative to have had additional demographic information, such as socioeconomic status. In the general population, socioeconomic status is associated with frailty, after controlling for race, age, and co-morbidities [71].

A future study might compare the level of frailty experienced by persons with IDD and in the general population to inform our understanding of aging with IDD. While this FI was created for persons with IDD, by emphasizing change in deficits and issues known to be related to frailty, it could easily be applied to the general population of home care users in Ontario, or elsewhere.

## Conclusion

Measuring frailty among persons with IDD using home care services is feasible. This study has identified health deficits applicable for those with IDD to include in a FI and has presented cut-off points for the FI to distinguish between risk groups.

Premature aging has frequently been reported in adults with IDD; however the increased vulnerabilities that come with aging are rarely quantified. Frailty may be a better way to understand the needs of the young old with IDD. In the general population, caring for elderly citizens is particularly challenging due to the blend of both medical and social problems [14], however adults with IDD face these challenges throughout their lives and these may worsen as they age.

Next steps include applying the FI to predictive models. If the FI is associated with time to adverse events (e.g. admission to LTC), the potential exists to use this measure as a tool in the community.

### Availability of supporting data

ICES is a prescribed entity under the Ontario Personal Health Information Protection Act. As such, ICES policies and procedures are approved by Ontario’s Information and Privacy Commissioner. These policies require that access to data be limited to persons who require such access to perform their role on an approved ICES Project or Third-Party Project. Thus, we are prohibited from making ICES data publicly available. Only the results of analysis of ICES data may be made available.

## Additional files

Additional file 1:
**Frailty index deficits, descriptions, and values.** A list of the deficits selected for inclusion into the frailty index. (DOCX 21 kb) Additional file 2:
**Deficits considered for inclusion in frailty index.** Deficits from the RAI-HC assessment considered for inclusion in the frailty index (FI) are presented alongside the results from the variable selection process in the tables below, by domain. Deficit items with no missing data (−) or had less than 30 % missing, with a prevalence greater than 5 % and less than 80 % (*), a correlation with age greater than r_s_ = +0.05 (*) and a significant association with health status (*p* < 0.05) (*) met the criteria. Bolded items indicate inclusion in the final FI. Italicized subheadings indicate categories within domains. (DOCX 59 kb) Additional file 3:
**Potential deficits within-category correlation.** The following tables include correlations (with Spearman correlation coefficients) between potential FI items that have already met the criteria presented in Appendix D (a significant, positive correlation with age; a prevalence between 5 and 80 %; less than 30 % missing data). Items are shown in categories with similar deficits. If a category had only one item meeting the criteria, the category is not presented as no correlations were calculated. (DOCX 23 kb) Additional file 4:
**Potential deficits within-category correlation.** Unadjusted odds ratio for 1-year admission to long-term care and the 42 FI items, sorted from strongest to weakest association. (DOCX 16 kb)

## Abbreviations

ADL: Activities of daily living
CAP: Clinical assessment protocol
CI: Confidence interval
CPS: Cognitive Performance Scale
FI: Frailty index
ICES: Institute for Clinical Evaluative Sciences
IDD: Intellectual and developmental disabilities
LTC: Long-term care
OR: Odds ratio
RAI-HC: Resident Assessment Instrument- Home Care
SD: Standard deviation
