# Supplementary material for: Using an accumulation of deficits approach to measure frailty in a population of home care users with intellectual and developmental disabilities: an analytical descriptive study
Source: BMC Geriatr. 2015 Dec 18;15:170. doi: 10.1186/s12877-015-0170-5 (PMC4683739; doi:10.1186/s12877-015-0170-5)
Supplement: Additional file 1: — Frailty index deficits, descriptions, and values. A list of the deficits selected for inclusion into the frailty index. (DOCX 21 kb) [file 12877_2015_170_MOESM1_ESM.docx]

Additional File 1: Frailty index deficits, descriptions, and values

| **Deficit Variable** | | **Description** | **FI Value** |
| --- | --- | --- | --- |
| ***Physiological*** | | | |
| 1 | Hearing impairment | Hears adequately  Minimal difficulty or in special situations only  Highly impaired | 0.0  0.5  1.0 |
| 2 | Cataract | Not present  Present (indicated by doctor) | 0.0  1.0 |
| 3 | ADL decline: Mobility in bed | Independent or no decline  Requires increased set up help, supervision or limited assistance  Requires increased extensive assistance, maximal support or completely dependent | 0.0  0.5  1.0 |
| 4 | ADL decline: Transfers/ in-home locomotion | Independent or no decline  Requires increased set up help, supervision or limited assistance  Requires increased extensive assistance, maximal support or completely dependent | 0.0  0.5  1.0 |
| 5 | ADL decline: Locomotion out of home | Independent or no decline  Requires increased set up help, supervision or limited assistance  Requires increased extensive assistance, maximal support or completely dependent | 0.0  0.5  1.0 |
| 6 | ADL decline: Dressing body | Independent or no decline  Requires increased set up help, supervision or limited assistance  Requires increased extensive assistance, maximal support or completely dependent | 0.0  0.5  1.0 |
| 7 | ADL decline: Hygiene and bathing | Independent or no decline  Requires increased set up help, supervision or limited assistance  Requires increased extensive assistance, maximal support or completely dependent | 0.0  0.5  1.0 |
| 8 | ADL decline: Toilet use | Independent or no decline  Requires increased set up help, supervision or limited assistance  Requires increased extensive assistance, maximal support or completely dependent | 0.0  0.5  1.0 |
| 9 | ADL decline: Eating | Independent or no decline  Requires increased set up help, supervision or limited assistance  Requires increased extensive assistance, maximal support or completely dependent | 0.0  0.5  1.0 |
| 10 | Stair climbing | Up and down stairs without help (last 3 days)  Up and down stairs with help or not at all (last 3 days) | 0.0  1.0 |
| 11 | Stamina | Leaves home every day (in a typical week)  Leaves home 2-6 days (in a typical week)  Leaves home once or never (in a typical week) | 0.0  0.5  1.0 |
| 12 | Fall frequency | No falls (last 90 days)  1-2 falls (last 90 days)  3-5 falls (last 90 days)  6-8 falls (last 90 days)  9 or more falls (last 90 days) | 0.0  0.25  0.50  0.75  1.0 |
| 13 | Unsteady gait | No unsteady gait  Unsteady gait | 0.0  1.0 |
| 14 | Stroke | Not present  Present (indicated by doctor) | 0.0  1.0 |
| 15 | Coronary artery disease | Not present  Present (indicated by doctor) | 0.0  1.0 |
| 16 | Hypertension | Not present  Present (indicated by doctor) | 0.0  1.0 |
| 17 | Other circulatory disease | Congestive heart failure, irregularly irregular pulse and peripheral vascular disease not present  Congestive heart failure, irregularly irregular pulse or peripheral vascular disease present (indicated by doctor) | 0.0  1.0 |
| 18 | Arthritis | Not present  Present (indicated by doctor) | 0.0  1.0 |
| 19 | Osteoporosis | Not present  Present (indicated by doctor) | 0.0  1.0 |
| 20 | Specific infection | HIV infection, pneumonia, tuberculosis and urinary tract infection not present  HIV infection, pneumonia, tuberculosis or urinary tract infection present (indicated by doctor) | 0.0  1.0 |
| 21 | Dementia/ Alzheimer’s | Not present  Present (indicated by doctor) | 0.0  1.0 |
| 22 | Diabetes | Not present  Present (indicated by doctor) | 0.0  1.0 |
| 23 | Respiratory disease | Emphysema, COPD and asthma not present  Emphysema, COPD or asthma present (indicated by doctor) | 0.0  1.0 |
| 24 | Worsening of continence | No worsening of bladder incontinence (last 90 days)  Worsening of bladder incontinence (last 90 days) | 0.0  1.0 |
| 25 | Edema | No edema (last 3 days)  Edema (last 3 days) | 0.0  1.0 |
| 26 | Shortness of breath | No shortness of breath (last 3 days)  Shortness of breath (last 3 days) | 0.0  1.0 |
| 27 | Pain frequency | No pain  Less than daily  At least once daily | 0.0  0.5  1.0 |
| 28 | Pain disruption | Pain does not disrupt usual activities  Pain disrupts usual activities | 0.0  1.0 |
| 29 | Number of medications | 0-3 different medications taken (last 7 days)  4-6 different medications taken (last 7 days)  7 or more different medications taken (last 7 days) | 0.0  0.5  1.0 |
| 30 | Use of antidepressant | Did not take antidepressant (last 7 days)  Used antidepressant (last 7 days) | 0.0  1.0 |
| ***Cognitive*** | | | |
| 31 | Short-term memory loss | Memory OK (appears to recall after 5 minutes)  Memory problem (does not appear to recall after 5 minutes) | 0.0  1.0 |
| 32 | Worsened decision-making | No worsening of decision making compared to 90 days ago  Worsening of decision making compared to 90 days ago | 0.0  1.0 |
| 33 | Delirium | No indicator of delirium (last 90 days)  Agitated or disoriented such that his/her safety is endangered | 0.0  1.0 |
| 34 | Communication decline | No worsening in communication compared to 90 days ago  Worsening in communication compared to 90 days ago | 0.0  1.0 |
| 35 | Changes in social activities | No decline in participation in social activities (last 90 days)  Decline in participation in social activities (last 90 days) | 0.0  1.0 |
| 36 | Social isolation | Never or hardly ever alone, or for about one hour, during the day  Alone for long periods of time or all the time | 0.0  1.0 |
| 37 | Loneliness | Does not feel lonely  Feels lonely | 0.0  1.0 |
| ***Psychological*** | | | |
| 38 | Mood decline | No worsening of mood indicators (last 90 days)  Worsening of mood indicators (last 90 days) | 0.0  1.0 |
| 39 | Changes in behaviour | No worsening of behavioural symptoms (last 90 days)  Worsening of behavioural symptoms (last 90 days | 0.0  1.0 |
| 40 | Fear of falling | No fear of falling preventing outdoor activity  Fear of falling limits outdoor activity | 0.0  1.0 |
| ***Service Utilization*** | | | |
| 41 | Hospital admission | No admission to hospital (with overnight stay) (last 90 days)  At least 1 admission to hospital (with overnight stay) (last 90 days) | 0.0  1.0 |
| 42 | Change in care needs | No change or improvement in overall self-sufficiency (last 90 days)  Deterioration in overall self-sufficiency (last 90 days) | 0.0  1.0 |
