# Supplementary material for: Using an accumulation of deficits approach to measure frailty in a population of home care users with intellectual and developmental disabilities: an analytical descriptive study
Source: BMC Geriatr. 2015 Dec 18;15:170. doi: 10.1186/s12877-015-0170-5 (PMC4683739; doi:10.1186/s12877-015-0170-5)
Supplement: Additional file 2: — Deficits considered for inclusion in frailty index. Deficits from the RAI-HC assessment considered for inclusion in the frailty index (FI) are presented alongside the results from the variable selection process in the tables below, by domain. Deficit items with no missing data (−) or had less than 30 % missing, with a prevalence greater than 5 % and less than 80 % (*), a correlation with age greater than rs = +0.05 (*) and a significant association with health status (p < 0.05) (*) met the criteria. Bolded items indicate inclusion in the final FI. Italicized subheadings indicate categories within domains. (DOCX 59 kb) [file 12877_2015_170_MOESM2_ESM.docx]

Additional File 2: Deficits considered for inclusion in frailty index

Deficits from the RAI-HC assessment considered for inclusion in the frailty index (FI) are presented alongside the results from the variable selection process in the tables below, by domain. Deficit items with no missing data (-) or had less than 30% missing, with a prevalence greater than 5% and less than 80% (*), a correlation with age greater than r_s_= +0.05 (*) and a significant association with health status (p<0.05) (*) met the criteria. Bolded items indicate inclusion in the final FI. Italicized subheadings indicate categories within domains.

Table D.1. Potential deficits from the physiological domain

| **Deficit** | **Missing (%)** | **Prevalence (%)** | | **Correlation with Age (r_s_)** | | **Association with Health Status (**𝛘**^2^)** | |
| --- | --- | --- | --- | --- | --- | --- | --- |
| *Sensory Impairments* | | | | | | | |
| **Hearing impairment** | **-** | **23.1** | ***** | **0.233** | ***** | **13.7** | ***** |
| Visual impairment | 0.01 | 25.7 | * | -0.010 |  | 22.5 | * |
| Visual Limitation/Difficulties | - | 1.6 |  | 0.043 |  | 49.0 | * |
| Vision Decline | - | 3.5 |  | 0.049 |  | 69.0 | * |
| **Cataract** | **-** | **6.7** | ***** | **0.175** | ***** | **6.1** | ***** |
| Glaucoma | - | 2.6 |  | 0.104 | * | 10.0 | * |
| *Activities of Daily Living* | | | | | | | |
| Help Needed with Mobility in Bed | 0.28 | 19.0 | * | -0.089 |  | 11.2 | * |
| **ADL Decline: Mobility in Bed** | **-** | **10.0** | ***** | **0.101** | ***** | **5.5** |  |
| Help Needed with Transferring | 0.15 | 30.4 | * | -0.079 |  | 19.3 | * |
| Help Needed with Locomotion in Home | 1.18 | 30.2 | * | -0.076 |  | 25.3 | * |
| **ADL Decline: Transfers/ In-home Locomotion** | **-** | **19.3** | ***** | **0.145** | ***** | **14.5** | ***** |
| Help Needed with Locomotion Out of Home | 14.56 | 36.1 | * | -0.130 |  | 20.2 | * |
| **ADL Decline: Locomotion Out of Home** | **-** | **15.5** | ***** | **0.096** | ***** | **29.0** | ***** |
| Help Needed Dressing Upper Body | 0.41 | 55.9 | * | -0.167 |  | 76.8 | * |
| Help Needed Dressing Lower Body | 0.89 | 58.5 | * | -0.158 |  | 53.5 | * |
| **ADL Decline: Dressing Body** | **-** | **28.6** | ***** | **0.170** | ***** | **9.8** | ***** |
| Help Needed Eating | 0.05 | 47.7 | * | -0.176 |  | 91.2 | * |
| **ADL Decline: Eating** | **-** | **22.2** | ***** | **0.137** | ***** | **5.2** | ***** |
| Help Needed with Toilet Use | 0.53 | 44.1 | * | -0.181 |  | 61.6 | * |
| **ADL Decline: Toilet Use** | **-** | **21.6** | ***** | **0.127** | ***** | **3.0** |  |
| Help Needed with Hygiene | 0.47 | 62.5 | * | -0.193 |  | 105.2 | * |
| Help Needed with Bathing | 1.78 | 79.1 | * | -0.111 |  | 30.1 | * |
| **ADL Decline: Hygiene and Bathing** | **-** | **38.1** | ***** | **0.211** | ***** | **58.6** | ***** |
| ADL Decline | - | 41.2 | * | 0.230 | * | 74.5 | * |
| *Mobility, Gait & Stamina* | | | | | | | |
| Primary Modes of Locomotion Indoors | 1.18 | 39.2 | * | 0.033 |  | 102.9 | * |
| Primary Modes of Locomotion Outdoors | 14.56 | 37.4 | * | -0.028 |  | 95.1 | * |
| **Stair Climbing** | **-** | **56.2** | ***** | **0.087** | ***** | **28.8** | ***** |
| **Stamina** | **-** | **68.7** | ***** | **0.196** | ***** | **59.0** | ***** |
| Physical Activity | - | 30.4 | * | 0.015 |  | 7.1 | * |
| Capable of Functional Independence (Client’s View) | - | 16.0 | * | 0.052 | * | 39.8 | * |
| Capable of Functional Independence (Caregiver’s View) | - | 10.3 | * | -0.025 |  | 83.7 | * |
| Good Prospects of Recovery | - | 8.1 | * | -0.020 |  | 4.9 | * |
| **Fall Frequency** | **-** | **34.3** | ***** | **0.142** | ***** | **78.9** | ***** |
| **Unsteady Gait** | **-** | **49.9** | ***** | **0.108** | ***** | **79.9** | ***** |
| *Incontinence* | | | | | | | |
| Bladder Incontinence | 0.17 | 46.0 | * | -0.061 |  | 24.3 | * |
| **Worsening of Bladder Incontinence** | **-** | **12.3** | ***** | **0.147** | ***** | **11.7** | ***** |
| Use of bladder device (pads or briefs) | - | 39.4 | * | -0.013 |  | 1.9 |  |
| Use of bladder device (urinary catheter) | - | 3.1 |  | 0.033 |  | 9.9 | * |
| Bowel Incontinence | 0.06 | 29.5 | * | -0.152 |  | 19.8 | * |
| *Cardiovascular Diagnoses* | | | | | | | |
| **Stroke** | **-** | **7.8** | ***** | **0.172** | ***** | **7.6** | ***** |
| Congestive Heart Failure | - | 4.4 |  | 0.141 | * | 39.0 | * |
| **Coronary Artery Disease** | **-** | **8.7** | ***** | **0.213** | ***** | **36.9** | ***** |
| **Hypertension** | **-** | **27.1** | ***** | **0.338** | ***** | **61.1** | ***** |
| Irregularly Irregular Pulse | - | 3.7 |  | 0.139 | * | 34.0 | * |
| Peripheral Vascular Disease | - | 2.8 |  | 0.066 | * | 14.0 | * |
| **Other Circulatory Disease** | **-** | **9.6** | ***** | **0.187** | ***** | **61.9** | ***** |
| *Neurological Diagnoses* | | | | | | | |
| Alzheimer’s Disease | - | 5.4 | * | 0.200 | * | 8.6 | * |
| Dementia (Not Alzheimer’s) | - | 11.3 | * | 0.278 | * | 6.8 | * |
| **Dementia/Alzheimer’s** | **-** | **16.3** | ***** | **0.351** | ***** | **17.3** | ***** |
| Head Trauma | - | 3.4 |  | -0.039 |  | 0.5 |  |
| Hemiplegia/Hemiparesis | - | 2.2 |  | -0.025 |  | 0.0 |  |
| Multiple Sclerosis | - | 0.6 |  | -0.039 |  | 2.0 | * |
| Parkinsonism | - | 2.2 |  | 0.117 | * | 13.0 | * |
| *Musculo-skeletal Diagnoses* | | | | | | | |
| **Arthritis** | **-** | **23.0** | ***** | **0.267** | ***** | **128.0** | ***** |
| Hip Fracture | - | 2.5 |  | 0.093 | * | 2.0 | * |
| Other Fracture | - | 6.9 | * | 0.073 | * | 0.3 |  |
| **Osteoporosis** | **-** | **10.6** | ***** | **0.185** | ***** | **6.4** | ***** |
| *Infectious Diagnoses* | | | | | | | |
| HIV Infection | - | 0.1 |  | -0.016 |  | 4.0 | * |
| Pneumonia | - | 3.0 |  | 0.004 |  | 3.0 |  |
| Tuberculosis | - | 0.0 |  | 0.001 |  | 4.0 |  |
| Urinary Tract Infection | - | 4.6 |  | 0.076 | * | 20.0 | * |
| **Specific Infection** | **-** | **7.4** | ***** | **0.060** | ***** | **23.1** | ***** |
| *Other Diagnoses* | | | | | | | |
| Cancer | - | 4.6 |  | 0.076 | * | 9.0 | * |
| **Diabetes** | **-** | **18.6** | ***** | **0.118** | ***** | **75.5** | ***** |
| **Respiratory Disease** | **-** | **11.9** | ***** | **0.064** | ***** | **97.4** | ***** |
| Renal Failure | - | 3.6 |  | 0.040 |  | 21.0 | * |
| Thyroid Disease | - | 13.8 | * | 0.075 | * | 0.1 |  |
| *Preventative Health Measures* | | | | | | | |
| Blood Pressure Taken | 4.97 | 78.5 | * | -0.001 |  | 0.2 |  |
| Influenza Vaccine Received | 4.97 | 57.5 | * | 0.097 | * | 0.6 |  |
| Colon Screening | 4.97 | 9.9 | * | 0.067 | * | 27.6 | * |
| Mammography or Breast Examination Received | 4.97 | 11.2 | * | 0.062 | * | 13.7 | * |
| *Problem Conditions* | | | | | | | |
| Diarrhea | - | 4.6 |  | -0.005 |  | 7.6 | * |
| Urination Difficulties | - | 4.6 |  | 0.070 | * | 97.0 | * |
| Fever | - | 0.5 |  | -0.018 |  | 17.0 | * |
| Loss of Appetite | - | 5.6 | * | 0.041 |  | 138.0 | * |
| Vomiting | - | 1.8 |  | -0.067 |  | 30.0 | * |
| Chest Pain | - | 2.4 |  | 0.024 |  | 119.0 | * |
| No Bowel Movement in 3 Days | - | 1.2 |  | -0.007 |  | 24.0 | * |
| Dizziness/Lightheadedness | - | 9.8 | * | 0.068 | * | 241.2 | * |
| **Edema** | **-** | **13.7** | ***** | **0.096** | ***** | **80.5** | ***** |
| **Shortness of Breath** | **-** | **12.7** | ***** | **0.086** | ***** | **261.2** | ***** |
| *Pain* | | | | | | | |
| **Pain Frequency** | **-** | **44.8** | ***** | **0.116** | ***** | **352.2** | ***** |
| Pain Intensity | - | 44.7 | * | 0.103 | * | 387.0 | * |
| **Pain Disruption** | **-** | **20.6** | ***** | **0.055** | ***** | **436.4** | ***** |
| Pain Character | - | 44.7 | * | 0.114 | * | 286.9 | * |
| Pain Medication | - | 16.0 | * | 0.018 |  | 222.1 | * |
| *Lifestyle Habits* | | | | | | | |
| Poor Drinking Habits | - | 2.1 |  | 0.047 |  | 28.0 | * |
| Drinking in Morning or Troubles | - | 0.9 |  | 0.026 |  | 12.0 | * |
| Daily Smoking | - | 12.9 | * | -0.010 |  | 97.0 | * |
| *Symptoms of Abuse* | | | | | | | |
| Fearful of Family Member or Caregiver | - | 0.8 |  | 0.013 |  | 7.8 | * |
| Unusually Poor Hygiene | - | 2.0 |  | 0.026 |  | 15.0 | * |
| Unexplained Injuries | - | 0.1 |  | 0.001 |  | 1.0 |  |
| Neglected or Abused | - | 0.7 |  | 0.018 |  | 0.3 |  |
| *Nutritional Status* | | | | | | | |
| Unintended Weight Loss | - | 7.0 | * | 0.048 |  | 44.0 | * |
| Severe Malnutrition | - | 1.3 |  | 0.009 |  | 14.0 | * |
| Morbid Obesity | - | 6.9 | * | -0.089 |  | 60.2 | * |
| Irregular Meals | - | 3.3 |  | 0.001 |  | 47.0 | * |
| Decreased Consumption | - | 2.4 |  | 0.024 |  | 33.0 | * |
| Insufficient Fluid | - | 1.3 |  | 0.008 |  | 6.0 | * |
| Enteral Tube Feeding | - | 3.1 |  | -0.178 |  | 9.0 | * |
| *Oral Health* | | | | | | | |
| Swallowing Difficulty | - | 18.0 | * | -0.148 |  | 21.5 | * |
| Chewing Problems | - | 11.7 | * | *-0.025* |  | 1.9 |  |
| *Oral Health cont’d* | | | | | | | |
| Dry Mouth While Eating | - | 1.3 |  | 0.011 |  | 23.0 | * |
| Brushing Teeth Difficulty | - | 9.9 | * | -0.101 |  | 12.2 | * |
| *Skin Conditions* | | | | | | | |
| Skin Problems | - | 26.5 | * | -0.004 |  | 5.0 | * |
| Pressure Ulcer | - | 4.6 |  | 0.008 |  | 1.4 |  |
| Stasis Ulcer | - | 1.9 |  | 0.024 |  | 2.0 |  |
| Burns | - | 0.2 |  | -0.021 |  | 0.9 |  |
| Open Lesions | - | 2.7 |  | -0.012 |  | 3.4 |  |
| Skin Tears/Cuts | - | 2.6 |  | 0.025 |  | 0.2 |  |
| Surgical Wounds | - | 4.4 |  | -0.051 |  | 2.8 |  |
| Skin Problems | - | 7.2 | * | 0.030 |  | 0.7 |  |
| History of Resolved Pressure Ulcer | - | 7.2 | * | -0.031 |  | 0.1 |  |
| Antibiotics for Ulcer/Wound | - | 4.5 |  | -0.006 |  | 0.9 |  |
| Dressings for Ulcer/Wound | - | 8.7 | * | -0.038 |  | 5.2 | * |
| Surgical Wound Care | - | 3.3 |  | -0.047 |  | 1.5 |  |
| Other Ulcer/Wound Care | - | 3.5 |  | -0.015 |  | 0.2 |  |
| *Medications* | | | | | | | |
| **Number of Medications** | **-** | **76.7** | ***** | **0.257** | ***** | **90.5** | ***** |
| Use of Antipsychotic/Neuroleptic | - | 34.9 | * | -0.017 |  | 1.6 |  |
| Use of Anxiolytic | - | 25.5 | * | -0.020 |  | 27.4 | * |
| **Use of Antidepressant** | **-** | **31.2** | ***** | **0.061** | ***** | **74.3** | ***** |
| Use of Hypnotic | - | 17.8 | * | 0.018 |  | 14.4 | * |
| Medications Have No Medical Oversight | 15.38 | 2.4 |  | -0.036 |  | 2.6 |  |
| Poor Compliance/Adherence to Meds | 3.6 | 10.3 | * | 0.050 | * | 44.2 | * |

Table D.2. Potential deficits from the cognitive domain

| **Deficit** | **Missing (%)** | **Prevalence (%)** | | **Correlation with Age (r_s_)** | | **Association with Health Status (**𝛘**^2^)** | |
| --- | --- | --- | --- | --- | --- | --- | --- |
| *Cognitive Patterns* | | | | | | | |
| **Short-Term Memory Loss** | **-** | **62.0** | ***** | **0.082** | ***** | **38.9** | ***** |
| Long-Term Memory Loss | - | 51.1 | * | -0.090 |  | 149.5 | * |
| Impaired Cognitive Skills for Daily Decision Making | - | 81.1 |  | -0.077 |  | 249.0 | * |
| **Worsening of Decision Making** | **-** | **16.7** | ***** | **0.231** | ***** | **14.7** | ***** |
| Change in Mental Function | - | 3.0 |  | 0.051 | * | 11.0 | * |
| **Delirium** | **-** | **8.7** | ***** | **0.070** | ***** | **2.8** |  |
| Making Self Understood | 0.03 | 55.7 | * | -0.204 |  | 205.6 | * |
| Ability to Understand Others | 0.01 | 56.9 | * | -0.159 |  | 204.1 | * |
| **Communication Decline** | **-** | **9.1** | ***** | **0.137** | ***** | **2.1** |  |
| *Instrumental Activities of Daily Living* | | | | | | | |
| Help Needed with Meal Preparation | 16.22 | 74.8 | * | -0.102 |  | 80.5 | * |
| Difficulty with Meal Preparation | - | 91.4 |  | -0.055 |  | 75.0 | * |
| Help Needed with Ordinary Housework | 16.89 | 76.7 | * | -0.079 |  | 20.6 | * |
| Difficulty with Ordinary Housework | - | 94.0 |  | -0.024 |  | 5.6 |  |
| Help Needed with Managing Finances | 16.75 | 71.9 | * | -0.147 |  | 156.5 | * |
| Difficulty with Managing Finances | - | 87.9 |  | -0.106 |  | 176.0 | * |
| Help Needed with Managing Medications | 19.99 | 64.2 | * | -0.144 |  | 155.2 | * |
| Difficulty with Managing Medications | - | 81.9 |  | -0.086 |  | 142.0 | * |
| Help Needed with Phone Use | 30.26 | 34.3 | * | -0.186 |  | 161.0 | * |
| Difficulty with Phone Use | - | 61.1 | * | -0.151 |  | 271.6 | * |
| Help Needed with Shopping | 20.03 | 72.4 | * | -0.087 |  | 37.0 | * |
| Difficulty with Shopping | - | 91.8 |  | -0.033 |  | 36.0 | * |
| Help Needed with Transportation | 20.21 | 58.9 | * | -0.133 |  | 46.6 | * |
| Difficulty with Transportation | - | 76.7 | * | -0.056 |  | 39.0 | * |
| *Help with Medical Equipment* | | | | | | | |
| Help Needed with Oxygen Equipment | 0.28 | 2.1 |  | -0.001 |  | 69.0 | * |
| Help Needed with IV Equipment | - | 1.4 |  | -0.009 |  | 18.0 | * |
| Help Needed with Catheter Equipment | - | 3.6 |  | 0.017 |  | 5.0 |  |
| Help Needed with Ostomy Equipment | - | 1.5 |  | -0.030 |  | 19.0 | * |

Table D.3. Potential deficits from the social domain

| **Deficit** | **Missing (%)** | **Prevalence (%)** | | **Correlation with Age (r_s_)** | | **Association with Health Status (**𝛘**^2^)** | |
| --- | --- | --- | --- | --- | --- | --- | --- |
| Withdrawal from Activities of Interest | - | 9.6 | * | 0.039 |  | 94.7 | * |
| Reduced Social Interaction | - | 12.7 | * | 0.027 |  | 88.3 | * |
| Not at Ease Interacting with Others | - | 12.0 | * | -0.064 |  | 4.4 | * |
| Openly Expresses Conflict/ Anger | - | 17.1 | * | -0.037 |  | 10.6 | * |
| **Changes in Social Activities** | **15.38** | **22.8** | ***** | **0.119** | ***** | **130.9** | ***** |
| **Social Isolation** | **15.38** | **25.5** | ***** | **0.196** | ***** | **95.9** | ***** |
| **Loneliness** | **15.38** | **9.4** | ***** | **0.079** | ***** | **194.0** | ***** |

Table D.4. Potential deficits from the psychological domain

| **Deficit** | **Missing (%)** | **Prevalence (%)** | | **Correlation with Age (r_s_)** | | **Association with Health Status (**𝛘**^2^)** | |
| --- | --- | --- | --- | --- | --- | --- | --- |
| *Mood* | | | | | | | |
| Feelings of Sadness or Being Depressed | - | 18.4 | * | 0.033 |  | 327.2 | * |
| Persistent Anger with Self or Others | - | 16.3 | * | -0.026 |  | 32.2 | * |
| Expressions of Apparently Unrealistic Fears | - | 8.2 | * | 0.009 |  | 90.0 | * |
| Repetitive Health Complaints | - | 6.4 | * | -0.004 |  | 287.0 | * |
| Repetitive Anxious Complains | - | 18.6 | * | -0.020 |  | 94.0 | * |
| Sad, Pained, Worried Facial Expressions | - | 20.9 | * | 0.040 |  | 295.3 | * |
| Recurrent Crying or Tearfulness | - | 11.5 | * | 0.003 |  | 88.0 | * |
| **Mood Decline** | **-** | **14.2** | ***** | **0.053** | ***** | **101.9** | ***** |
| *Behaviour Symptoms* | | | | | | | |
| Wandering Behaviour | - | 5.2 | * | 0.011 |  | 17.0 | * |
| Verbally Abusive Behavioural Symptoms | - | 8.6 | * | 0.002 |  | 0.5 |  |
| Physically Abusive Behavioural Symptoms | - | 4.5 |  | -0.097 |  | 21.0 | * |
| Socially Inappropriate/Disruptive Behavioural Symptoms | - | 10.4 | * | *-0.126* |  | 32.0 | * |
| Resists Care | - | 12.8 | * | -0.021 |  | 8.1 | * |
| **Changes in Behaviour** | **-** | **10.1** | ***** | **0.064** | ***** | **0.1** |  |
| *Other Psychological Symptoms* | | | | | | | |
| **Fear of Falling** | **-** | **29.3** | ***** | **0.094** | ***** | **118.0** | ***** |
| Delusions | - | 3.8 |  | 0.052 | * | 4.1 | * |
| Hallucinations | - | 4.4 |  | 0.023 |  | 9.3 | * |
| Psychological Disorder |  | 32.6 | * | 0.012 |  | 104.4 | * |

Table D.5. Potential deficits from the environment/service use domain

| **Deficit** | **Missing (%)** | **Prevalence (%)** | | **Correlation with Age (r_s_)** | | **Association with Health Status (**𝛘**^2^)** | |
| --- | --- | --- | --- | --- | --- | --- | --- |
| *Home Environment* | | | | | | | |
| Hazardous/Uninhabitable Lighting | 4.97 | 0.5 |  | -0.011 |  | 17.0 | * |
| Hazardous/Uninhabitable Flooring/Carpet | 4.97 | 1.9 |  | 0.009 |  | 13.4 | * |
| Hazardous/Uninhabitable Bathroom | - | 2.7 |  | -0.008 |  | 8.0 | * |
| Hazardous/Uninhabitable Kitchen | - | 0.7 |  | 0.006 |  | 0.0 |  |
| Hazardous/Uninhabitable Heating/Cooling | - | 0.6 |  | 0.017 |  | 1.8 |  |
| Home Environment Threatens Personal Safety | - | 1.8 |  | 0.025 |  | 10.0 | * |
| Difficulty Accessing Home | - | 6.3 | * | 0.039 |  | 28.5 | * |
| Difficulty Accessing Rooms in House | 4.97 | 5.5 | * | -0.044 |  | 3.7 |  |
| Client lives with others | - | 9.6 | * | 0.000 |  | 5.0 | * |
| *Service Utilization* | | | | | | | |
| **Hospital Admissions** | **-** | **36.0** | ***** | **0.132** | ***** | **56.7** | ***** |
| Number of Visits to Emergency | - | 21.6 | * | 0.036 |  | 113.2 | * |
| Number of Emergent Care Visits | 15.38 | 5.2 | * | -0.005 |  | 20.7 | * |
| Unmet Treatment Goals | 15.38 | 9.1 | * | 0.026 |  | 2.1 |  |
| **Change in Care Needs** | **-** | **45.4** | ***** | **0.232** | ***** | **53.3** | ***** |
| Made Health Trade-Offs | 15.38 | 2.3 |  | -0.023 |  | 82.8 | * |
