# Supplementary material for: Using an accumulation of deficits approach to measure frailty in a population of home care users with intellectual and developmental disabilities: an analytical descriptive study
Source: BMC Geriatr. 2015 Dec 18;15:170. doi: 10.1186/s12877-015-0170-5 (PMC4683739; doi:10.1186/s12877-015-0170-5)
Supplement: Additional file 3: — Potential deficits within-category correlation. The following tables include correlations (with Spearman correlation coefficients) between potential FI items that have already met the criteria presented in Appendix D (a significant, positive correlation with age; a prevalence between 5 and 80 %; less than 30 % missing data). Items are shown in categories with similar deficits. If a category had only one item meeting the criteria, the category is not presented as no correlations were calculated. (DOCX 23 kb) [file 12877_2015_170_MOESM3_ESM.docx]

**Additional File 3: Potential deficits within-category correlation**

The following tables include correlations (with Spearman correlation coefficients) between potential FI items that have already met the criteria presented in Appendix D (a significant, positive correlation with age; a prevalence between 5 and 80%; less than 30% missing data). Items are shown in categories with similar deficits. If a category had only one item meeting the criteria, the category is not presented as no correlations were calculated.

Table E.1. Correlation matrix: Sensory impairment

|  |  | **1** | **2** |
| --- | --- | --- | --- |
| **1** | **Hearing Impairment** | 1.00 | 0.09 |
| **2** | **Cataract** |  | 1.00 |

Table E.2. Correlation matrix: Activities of daily living

|  |  | **1** | **2** | **3** | **4** | **5** | **6** | **7** |
| --- | --- | --- | --- | --- | --- | --- | --- | --- |
| **1** | **ADL Decline: Mobility in Bed** | 1.00 | 0.68 | 0.29 | 0.56 | 0.56 | 0.61 | 0.49 |
| **2** | **ADL Decline: Transfers / In-home Locomotion** |  | 1.00 | 0.47 | 0.74 | 0.69 | 0.78 | 0.68 |
| **3** | **ADL Decline: Locomotion Out of Home** |  |  | 1.00 | 0.53 | 0.47 | 0.45 | 0.53 |
| **4** | **ADL Decline: Dressing Body** |  |  |  | 1.00 | 0.77 | 0.80 | 0.83 |
| **5** | **ADL Decline: Eating** |  |  |  |  | 1.00 | 0.76 | 0.73 |
| **6** | **ADL Decline: Toilet Use** |  |  |  |  |  | 1.00 | 0.74 |
| **7** | **ADL Decline: Hygiene and Bathing** |  |  |  |  |  |  | 1.00 |

Table E.3. Correlation matrix: Mobility, gait, and stamina

|  |  | **1** | **2** | **3** | **4** | **5** |
| --- | --- | --- | --- | --- | --- | --- |
| **1** | **Stair Climbing** | 1.00 | 0.39 | 0.08 | 0.18 | 0.39 |
| **2** | **Stamina** |  | 1.00 | 0.08 | 0.11 | 0.24 |
| **3** | **Capable of Functional Independence (Client's View)** |  |  | 1.00 | 0.10 | 0.07 |
| **4** | **Fall Frequency** |  |  |  | 1.00 | 0.38 |
| **5** | **Unsteady Gait** |  |  |  |  | 1.00 |

Table E.4. Correlation matrix: Cardiovascular diagnoses

|  |  | **1** | **2** | **3** | **4** |
| --- | --- | --- | --- | --- | --- |
| **1** | **Stroke** | 1.00 | 0.14 | 0.16 | 0.10 |
| **2** | **Coronary Artery Disease** |  | 1.00 | 0.22 | 0.18 |
| **3** | **Hypertension** |  |  | 1.00 | 0.18 |
| **4** | **Other Circulatory Disease** |  |  |  | 1.00 |

Table E.5. Correlation matrix: Musclo-skeletal diagnoses

|  |  | **1** | **2** | **3** |
| --- | --- | --- | --- | --- |
| **1** | **Arthritis** | 1.00 | 0.04 | 0.16 |
| **2** | **Other Fracture** |  | 1.00 | 0.11 |
| **3** | **Osteoporosis** |  |  | 1.00 |

Table E.6. Correlation matrix: Other diagnoses

|  |  | **1** | **2** | **3** |
| --- | --- | --- | --- | --- |
| **1** | **Diabetes** | 1.00 | 0.07 | 0.04 |
| **2** | **Respiratory Disease** |  | 1.00 | 0.04 |
| **3** | **Thyroid Disease** |  |  | 1.00 |

Table E.7. Correlation matrix: Problem conditions

|  |  | **1** | **2** | **3** |
| --- | --- | --- | --- | --- |
| **1** | **Dizziness/ Lightheadedness** | 1.00 | 0.10 | 0.20 |
| **2** | **Edema** |  | 1.00 | 0.19 |
| **3** | **Shortness of Breath** |  |  | 1.00 |

Table E.8. Correlation matrix: Preventative health measures

|  |  | **1** | **2** | **3** |
| --- | --- | --- | --- | --- |
| **1** | **Influenza Vaccine Received** | 1.00 | 0.15 | 0.15 |
| **2** | **Colon Screening** |  | 1.00 | 0.21 |
| **3** | **Mammography or Breast Examination Received** |  |  | 1.00 |

Table E.9. Correlation matrix: Pain

|  |  | **1** | **2** | **3** | **4** |
| --- | --- | --- | --- | --- | --- |
| **1** | **Pain Frequency** | 1.00 | 0.95 | 0.61 | 0.96 |
| **2** | **Pain Intensity** |  | 1.00 | 0.61 | 0.98 |
| **3** | **Pain Disruption** |  |  | 1.00 | 0.57 |
| **4** | **Pain Character** |  |  |  | 1.00 |

Table E.10. Correlation matrix: Medications

|  |  | **1** | **2** | **3** |
| --- | --- | --- | --- | --- |
| **1** | **Number of Medications** | 1.00 | 0.28 | 0.00 |
| **2** | **Use of Antidepressants** |  | 1.00 | 0.03 |
| **3** | **Poor Compliance/ Adherence to Meds** |  |  | 1.00 |

Table E.11. Correlation matrix: Cognitive patterns

|  |  | **1** | **2** | **3** | **4** |
| --- | --- | --- | --- | --- | --- |
| **1** | **Short-Term Memory Loss** | 1.00 | 0.95 | 0.61 | 0.96 |
| **2** | **Worsening of Decision Making** |  | 1.00 | 0.61 | 0.98 |
| **3** | **Delirium** |  |  | 1.00 | 0.57 |
| **4** | **Communication decline** |  |  |  | 1.00 |

Table E.12. Correlation matrix: Social domain

|  |  | **1** | **2** | **3** |
| --- | --- | --- | --- | --- |
| **1** | **Changes in Social Activities** | 1.00 | 0.08 | 0.11 |
| **2** | **Social Isolation** |  | 1.00 | 0.28 |
| **3** | **Loneliness** |  |  | 1.00 |

Table E.13. Correlation matrix: Service utilization

|  |  | **1** | **2** |
| --- | --- | --- | --- |
| **1** | **Hospital Admissions** | 1.00 | 0.35 |
| **2** | **Change in Care Needs** |  | 1.00 |
