# Supplementary material for: Using an accumulation of deficits approach to measure frailty in a population of home care users with intellectual and developmental disabilities: an analytical descriptive study
Source: BMC Geriatr. 2015 Dec 18;15:170. doi: 10.1186/s12877-015-0170-5 (PMC4683739; doi:10.1186/s12877-015-0170-5)
Supplement: Additional file 4: — Potential deficits within-category correlation. Unadjusted odds ratio for 1-year admission to long-term care and the 42 FI items, sorted from strongest to weakest association. (DOCX 16 kb) [file 12877_2015_170_MOESM4_ESM.docx]

Additional File 4: Odds of long-term care by deficit

Unadjusted odds ratio for 1-year admission to long-term care and the 42 FI items, sorted from strongest to weakest association

| **FI Item** | **OR (95% CI)** | **p-value** |
| --- | --- | --- |
| *FI (Frail vs. Non-Frail)* | *4.45 (3.82-5.19)* | *<.0001* |
| ADL decline: toilet use | 3.62 (3.06-4.28) | <.0001 |
| ADL decline: locomotion out of home | 3.61 (3.02-4.33) | <.0001 |
| ADL decline: dressing body | 3.44 (2.93-4.03) | <.0001 |
| ADL decline: hygiene and bathing | 3.41 (2.96-3.92) | <.0001 |
| Change in care needs | 3.06 (2.67-3.51) | <.0001 |
| Worsened decision-making | 3.02 (2.61-3.50) | <.0001 |
| Communication decline | 2.94 (2.46-3.52) | <.0001 |
| ADL decline: mobility in bed | 2.82 (2.18-3.65) | <.0001 |
| Worsening of continence | 2.75 (2.34-3.24) | <.0001 |
| Dementia/Alzheimer's | 2.63 (2.27-3.05) | <.0001 |
| Stamina | 2.41 (2.12-2.75) | <.0001 |
| Stair climbing | 2.37 (2.05-2.73) | <.0001 |
| Delirium | 2.27 (1.88-2.75) | <.0001 |
| Hospital admission | 2.00 (1.75-2.27) | <.0001 |
| Number of medications | 1.94 (1.70-2.22) | <.0001 |
| Changes in behaviour | 1.93 (1.60-2.32) | <.0001 |
| Stroke | 1.92 (1.57-2.35) | <.0001 |
| Short-term memory loss | 1.91 (1.65-2.20) | <.0001 |
| Unsteady gait | 1.83 (1.60-2.09) | <.0001 |
| ADL decline: eating | 1.82 (1.35-2.44) | <.0001 |
| ADL decline: transfers/ in-home locomotion | 1.76 (1.36-2.27) | <.0001 |
| Specific infection | 1.69 (1.36-2.10) | <.0001 |
| Coronary artery disease | 1.61 (1.32-1.97) | <.0001 |
| Hypertension | 1.56 (1.36-1.79) | <.0001 |
| Mood decline | 1.56 (1.32-1.85) | <.0001 |
| Fall frequency | 1.56 (1.30-1.87) | <.0001 |
| Change in social activities | 1.47 (1.24-1.75) | <.0001 |
| Cataract | 1.45 (1.15-1.83) | 0.0019 |
| Other circulatory disease | 1.44 (1.18-1.76) | 0.0003 |
| Osteoporosis | 1.42 (1.18-1.72) | 0.0003 |
| Fear of falling | 1.39 (1.22-1.60) | <.0001 |
| Loneliness | 1.32 (1.04-1.68) | 0.0227 |
| Edema | 1.27 (1.07-1.52) | 0.0077 |
| Arthritis | 1.15 (0.99-1.34) | 0.0612 |
| Diabetes | 1.12 (0.95-1.31) | 0.187 |
| Pain frequency | 1.11 (0.97-1.28) | 0.1237 |
| Use of antidepressant | 1.09 (0.95-1.25) | 0.2138 |
| Shortness of breath | 1.06 (0.87-1.28) | 0.5671 |
| Hearing impairment | 1.05 (0.67-1.64) | 0.8448 |
| Social isolation | 1.02 (0.86-1.22) | 0.8251 |
| Pain disruption | 1.01 (0.86-1.18) | 0.9417 |
| Respiratory disease | 0.91 (0.75-1.12) | 0.3841 |
